# The Effect of Different Freshness of Raw Material on Lipid Quality and Sensory Acceptance of Canned Sardines

**DOI:** 10.3390/foods11131987

**Published:** 2022-07-05

**Authors:** Zuzana Reblová, Santiago P. Aubourg, Jan Pokorný

**Affiliations:** 1 Faculty of Food and Biochemical Technology, University of Chemistry and Technology, Prague, Technická 5, Dejvice, 166 28 Prague, Czech Republic; zuzana.reblova@vscht.cz (Z.R.); jan.pokorny@vscht.cz (J.P.); 2 Marine Research Institute (CSIC), c/E. Cabello, 6, 36208 Vigo, Spain

**Keywords:** sardine, storage on ice, freshness, canning, acyl glycerols, fatty acids, ω3 PUFA, browning, sensory acceptance

## Abstract

We studied how storing fresh sardines (*Sardina pilchardus*) on ice for 0–15 days would affect lipid quality and sensory acceptance after the sardines were later canned. Average moisture and diacylglycerol contents showed a decreasing trend during storage time for sardines stored for to 0–10 days and an increasing trend for samples stored for 13–15 days. Total lipid and triacylglycerol average values increased with storage time of 0–10 days. In contrast, sardines stored for 13–15 days showed decreased values of lipids and triacylglycerols. Increased storage times also led to increased average saturated fatty acid (STFA) content and browning and decreased polyunsaturated fatty acid (PUFA) values and PUFA/STFA and ω3/ω6 ratios. Notably, the effect of storage time on PUFA/STFA and ω3/ω6 ratios and browning development was found significant (*p* < 0.05). Sensory descriptors revealed only slight quality differences with previous storage on ice for 0-10 days. In contrast, a substantial (*p* < 0.05) decrease (appearance and texture) was detected in samples corresponding to a 13–15-day period, such samples being considered unacceptable. Storage on ice not exceeding 10 days is recommended for sardines before being shipped to canneries for further processing. Furthermore, the use of efficient storage including preserving technologies would be desirable.

## 1. Introduction

Canned fish and invertebrate marine species are products of great economic importance in many countries and represent one of the most important and traditional means of seafood preservation [1]. In it, heat treatment involved (i.e., cooking and sterilisation) can substantially alter the nature of the raw starting material and provide a food product with different characteristics and supporting an important role in human nutrition [2,3]. Both enzymes and bacteria should be permanently inactivated as a result of heat treatment and provided reinfection does not occur and no negative interaction with the container is produced, canned seafood should keep for a very long time [4,5].

However, canneries need to store the raw material before it is canned or transported to factory. Remarkably, the time elapsed after being caught or harvested till the moment of processing can be a decisive period for the quality of the final canned product. Therefore, the majority of quality problems found in canned products can be related to the quality of the raw material, which continuously changes during storage [6,7]. As storage conditions, two main strategies have been employed in countries where a developed technology is available, namely refrigerated and frozen storage. Although frozen storage has been the most used method, storage on ice has been used commonly when a long storage period is not necessary [8,9]. Thus, storage on ice has shown great advantages as a cooling process since it partially inhibits most damage mechanisms (i.e., endogenous enzyme activity, microbial decomposition, and lipid oxidation) and retains fish quality for a reasonable time [10,11].

Since marine lipid composition is highly unsaturated, lipid damage during the thermal treatment of canning is likely to occur, especially if the material has been previously refrigerated. This damage mechanism can be enhanced if relatively long periods of refrigerated storage are required or if storage temperature is not properly maintained through the complete distribution chain before delivery to the cannery [8,12]. Previous research concerning the effect of refrigeration storage on the quality of canned seafood is scarce and focused on proximate composition [13], lipid hydrolysis and oxidation development [14], formation of fluorescent compounds [15], and sensory acceptance [16,17]. However, no information is available, to the best of our knowledge, concerning the effect of previous storage on ice on changes related to lipid class and fatty acid (FA) composition of canned seafood.

The present research focused on the effect of storage time on ice of fish prior to canning on lipid quality and sensory acceptance of the resulting canned product. For it, European sardines (*Sardina pilchardus*) individuals were stored on ice for different times (0–15 days) and subjected to canning process. After a 10-month storage, canned products were opened and the sardine muscle subjected to chemical composition (moisture and lipid contents, lipid class values, and FA profile), browning development and sensory acceptance (appearance, odour and texture) analyses.

## 2. Materials and Methods

### 2.1. Raw Fish, Storage on Ice, and Canning

Fresh European sardines (*S. pilchardus*) (72 specimens) were purchased at Vigo harbour (Northwestern Spain). The length of the specimens was in the 18–22 cm range and the average weight was 160 g. Whole sardines were kept on ice (0.5 °C) in an isothermal room (3–4 °C) for 15 days in three independent experimental sets (*n* = 3). Ice was prepared with an Icematic F100 Compact device (Castelmac SPA, Castelfranco, Italy). Fish specimens were surrounded by ice at a 1:1 fish to ice ratio; when required, ice was renewed. On defined days (0, 2, 6, 10, 13, and 15 days), individual sardines were withdrawn and steam cooked in our pilot plant (102–103 °C) to a final backbone temperature of 65 °C [18]. The fish were then cooled at room temperature (14 °C) for about three hours, beheaded, eviscerated, and filleted.

Fillets corresponding to two individual sardines were placed in each small rectangular can (105 × 60 × 25 mm; 150 mL). Brine (aq. 2% NaCl) was employed as filling medium. The cans were vacuum sealed and sterilised in a retort (115 °C, 45 min; *F_o_* = 7 min); such lethality value was chosen according to previous research [2]. After 10 months of storage at room temperature (18–20 °C), the cans were opened, one half of them being employed for the chemical analysis while the other half was used for the sensory analysis.

A 15-day storage period was considered useful to check the effect on lipid values and sensory acceptance in the current study. Additionally, a brine filling medium was found to be accurate to avoid possible interactions of the sardine lipids with a filling medium including a lipid component (i.e., vegetable oil).

### 2.2. Chemical Analyses

Once opened, the liquid part of the can was carefully drained off gravimetrically and filtered through a filter paper. Then, the sardine white muscle was separated, wrapped in filter paper, and used for analysis.

Moisture content was determined by weight difference in homogenised canned muscle (2–5 g) before and after 4 h at 105 °C [19]. Results were expressed as g·kg^−1^ muscle.

Lipid fraction was extracted from the fish muscle by the Folch et al. [20] method. For it, a mixture of fish muscle and extracting solvent (chloroform-methanol, 2:1) (1:20, tissue/solvent ratio) was homogenised, filtered, and centrifuged (3500× *g* for 10 min at 4 °C). The upper phase was removed, the resulting organic solution being washed by shaking with an aq. NaCl solution (0.5%) (1:4, salt solution-lipid extract ratio). After centrifugation (3500× *g* for 10 min at 4 °C), the lipid extract was recovered and diluted to a final volume by addition of 2:1 chloroform–methanol mixture.

For quantitative purposes, an aliquot of the lipid extract was smoothly warmed (ca. 40 °C) and carried to a constant weight, according to the Herbes and Hallen [21] procedure. Results were expressed as g·kg^−1^ muscle.

Assessment of TAG and DAG values was determined in the lipid extract from canned fish muscle by high-pressure size exclusion chromatography (HP-SEC) [22]. The system consisted of a high-pressure pump LCP 4000 (Ecom, Prague, Czech Republic), an HP 1050 series autosampler, and an HP 1047A series refractometric detector (Agilent Technologies, Santa Clara, CA, USA). The chromatographic separation was performed using a PL gel MIXED-E SEC column (7.5 mm × 300 mm, 3 μm) equipped with a guard column (7.5 mm × 50 mm, 5 μm, Agilent Technologies). Tetrahydrofuran was used as the mobile phase at a flow rate 0.6 mL·min^−1^; the injection volume was 5 μL. The injection-to-injection run time was 22 min. The temperature of the detector was kept at 30 °C. The TAG and DAG contents were quantified using the internal normalisation method.

Lipid extracts were converted into FA methyl esters (FAME) by using acetyl chloride in methanol and then analysed using a Perkin-Elmer 8700 gas chromatograph (Madrid, Spain) equipped with a fused silica capillary column SP-2330 (0.25 mm i.d. × 30 m, 0.20 μm film, Supelco Inc., Bellefonte, PA, USA) [16]. Peaks corresponding to FAME were identified by comparing their retention times with those of standard mixtures (Qualmix Fish, Larodan, Malmo, Sweden; FAME mix, Supelco, Inc., Bellefonte, PA, USA). Peak areas were automatically integrated; C19:0 FA was used as the internal standard for quantitative purposes. Content of each FA was calculated as g·100 g^−1^ total FA. Such values were employed to obtain the content on FA groups (saturated FA, STFA; monounsaturated FA, MUFA; polyunsaturated FA, PUFA) (g·100 g^−1^ total FA) as well as the total PUFA/total STFA and total ω3/total ω6 ratios.

The brown colour formation (BCF) in sardine muscle was determined spectrophotometrically (Beckman Coulter DU 640, Spectrophotometer, Beckman Coulter Inc., Brea, CA, USA) in the lipid extract at 420 nm according to Labuza and Massaro [23]. Results were calculated using the following formula: BCF = *A* × *V*/*w*, where *A* is the absorbance reading, and *V* and *w* denote the volume (mL) and the mass (mg), respectively, of the lipid extract measured.

### 2.3. Sensory Analysis

The sensory analysis was conducted according to the Quality Descriptive Analysis (QDA) method by a sensory panel consisting of 10 experienced judges (5 females and 5 males) selected and trained according to international standards in use of descriptors for raw and processed fish of different quality conditions [17,24]. As a first step of the analysis, the cans were opened, the whole contents being examined for appearance (brown colour in solid and liquid fractions; turbidity in liquid fraction; fish muscle shredding) and odour (presence of off-odours). Then, the fish muscle was taken out of the cans, cut into halves and examined for texture (resistance of muscle to be deformed).

Samples were presented to panelists in individual trays and were scored individually. The panel members shared samples tested. The different sensory descriptors were evaluated on non-structured linear scales with numerical scores included in the 0–100 range. For all descriptors, score 0 corresponds to the stage where such properties are observed in their maximum quality value and no further increase is possible, while score 100 represents the stage where a quality decrease is no more noticeable. Scores among panelists were averaged. For all descriptors, score 50 was considered the borderline of acceptability.

### 2.4. Statistical Analysis

Data obtained from chemical and sensory analyses were subjected to the ANOVA method to explore differences resulting from the effect of the previous storage time on ice. The comparison of means was performed using the least-squares difference (LSD) method. In all cases, analyses were carried out using the PASW Statistics 18 software for Windows (SPSS Inc., Chicago, IL, USA); differences were considered significant for a confidence interval at the 95% level (*p* < 0.05) in all cases. Correlation values among chemical values, sensory scores, and previous storage time were analysed by the Pearson test.

## 3. Results and Discussion

### 3.1. Determination of Moisture and Lipid Contents

Moisture content in canned sardines was included in the 687.8–725.2 g·kg^−1^ muscle range (Table 1). Values can be considered similar to those reported in canned sardines [25,26]. According to previous related research, average values of current canned fish did not provide a general trend with storage time before canning [15]. Thus, a decreasing tendency could be observed for samples corresponding to the 0–10-day period that was followed by an increasing trend for fish muscle subjected to the 13–15-day storage. Thus, the lowest average values were detected in canned sardines corresponding to 6 and 10 days of storage in ice before canning. In contrast, the highest average values were found in canned muscle subjected before canning to storage on ice of 0 and 15 days.

Different factors can be involved in changes of moisture content in the present study. On one side, protein damage during storage on ice and the canning process can lead to a water holding capacity decrease so that a moisture loss in muscle can be produced. On the other side, storage on ice and the canning process can lead to degradation and therefore loss of other muscle constituents, which in turn may lead to a relative increase of moisture content in muscle. Results obtained in the present study can be considered the result of both effects, different trends being explained on the basis of the relative importance of such effects.

Current data on lipid content (48.8–68.4 g·kg^−1^ muscle; Table 1) can be considered high when compared to previous studies on canned sardines [15,16]. Remarkably, varying values for lipid content in canned sardines have been reported according to different factors such as the packing medium, the particular species encountered, and the catching season [13,15,25,26]. As for moisture content, a general trend with previous storage time could not be concluded for lipid content values in agreement with previous related research [15]. Thus, an increasing tendency was detected in canned fish corresponding to a previous 0–10-day storage time, which was followed by a decreased content in fish previously stored for a 13–15-day period. As a result, the highest average value was detected in canned muscle that was previously stored for 10 days, while the lowest average value was found in canned fish from 0-day storage; remarkably, differences between both values were found significant (*p* < 0.05). According to previous studies on canned fish and seafood in general, an inverse ratio between lipid and moisture contents was detected in the present study [9,27].

Present results on lipid contents can be considered the result of two opposite effects. On one side, storage on ice and the canning process can lead to lipid oxidation development, which in turn may lead to the formation of lower molecular-weight molecules, susceptible to be lost in the hydrophilic filling medium employed during canning. On the other side, storage on ice, and the canning process especially, can facilitate protein denaturation, leading to a water-holding capacity loss of canned fish muscle so that a moisture decrease can be produced and consequently a relative lipid content increase.

### 3.2. Fatty Acid (FA) Analysis

The FA composition of canned sardines is shown in Table 2. The most abundant FA were C22:6ω3, C16:0, and C20:5ω3, according to previous research on the current species and fish in general [16,27].

Other remarkable FA constituents present in the canned muscle were C20:4ω6, C18:0, and C18:1ω9. As expressed in Table 2, no significant differences (*p* > 0.05) were detected in individual FA contents with previous storage time.

Average values of FA groups (STFA, MUFA, and PUFA) revealed slight differences as a result of previous storage time (Table 2); thus, a decreasing content for PUFA presence (*r* = −0.84) and an increasing presence for STFA (*r* = 0.83) were detected with previous storage time on ice. In the case of the MUFA group, a definite trend could not be concluded although the lowest average value was observed in canned fish corresponding to a previous 15-day storage. In spite of such tendencies found, and according to Table 2, no significant differences (*p* > 0.05) could be detected for the FA groups with previous storage time. In contrast, comparison of the contents of the different FA groups for each kind of canned sample revealed great differences (Table 2). Thus, PUFA showed to be the most abundant group (52.3–54.5 g·100 g^−1^ total FA) (*p* < 0.05), while MUFA provided the lowest values (10.4–12.6 g·100 g^−1^ total FA) (*p* > 0.05) and STFA revealed an intermediate range (32.6–35.1 g·100 g^−1^ total FA).

**Figure 1:**
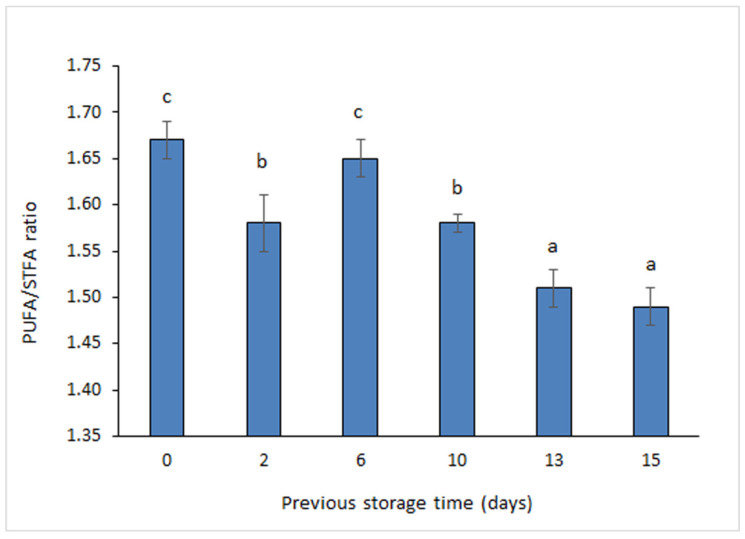
Determination of the polyunsaturated fatty acid/saturated fatty acid ratio in canned sardines subjected previously to storage on ice. Average values of three replicates (*n* = 3). Standard deviations are indicated by bars. Average values accompanied by different letters (a–c) denote significant differences (*p* < 0.05).

The PUFA/STFA ratio has shown to be a useful tool for checking the oxidation degree of marine fats and oils [28,29]. In the present study, a decreasing trend was detected for this ratio by increasing the previous storage time (*r* = −0.84) (Figure 1); thus, the lowest values (*p* < 0.05) were found in canned fish corresponding to the longest storage times (i.e., 13 and 15 days), while the highest average score was obtained in sardines canned at day 0. Therefore, an increased lipid damage development in canned sardine muscle was concluded with previous storage time.

A similar trend was detected in previous related research when analysing the polyene index (PI) [15], calculated as the following FA concentration ratio: (C22:6ω3 + C20:5ω3)/C16:0; in that study, a PI decrease was found in brine-canned sardines (*S. pilchardus*) by increasing the previous holding time. Additionally, a decrease of the PI was detected in sunflower oil-canned sardines (*S. pilchardus*) as a result of a 2-day holding time in flake ice [16]. In contrast, Rodríguez et al. [30] did not detect variation of the PI in canned salmon (*Oncorhynchus kisutch*) if previously stored in slurry ice for 5 and 9 days.

Seafood lipids deteriorate during refrigeration due to the development of different damage pathways such as enzymatic and non-enzymatic lipid oxidation [10,11]. Present research has shown a marked effect of previous storage time on PUFA/SAT ratio in the canned muscle of sardines. The decrease of this ratio can be explained on the basis of lipid damage development during previous storage. According to previous research, lipid oxidation and hydrolysis have been reported to be produced during the storage on ice of fatty fish species and lead to remarkable losses of nutritional and sensory values [12,31]. Remarkably, lipid oxidation development in raw fish would enhance the development of such a damage pathway during the subsequent thermal treatment (i.e., canning process) [8,14,16].

**Figure 2:**
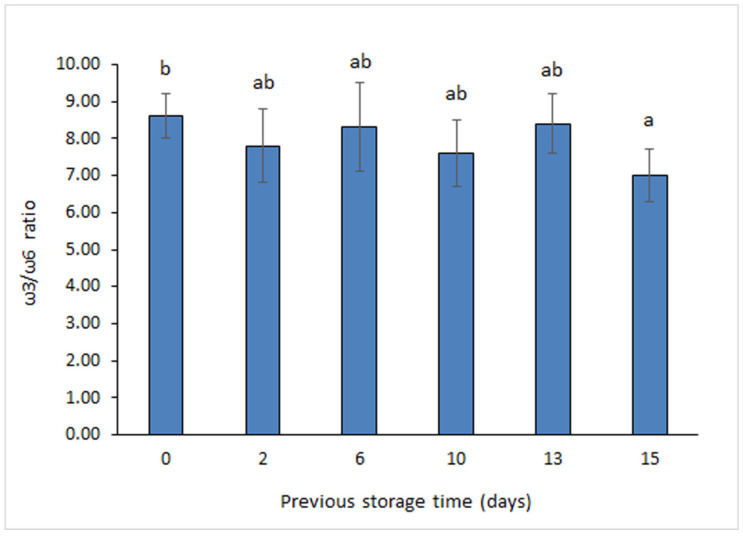
Determination of the ω3/ω6 ratio in canned sardines subjected previously to storage on ice. Average values of three replicates (*n* = 3). Standard deviations are indicated by bars. Average values accompanied by different lowercase letters (a,b) denote significant differences (*p* < 0.05).

Concerning PUFA series, great attention has been accorded to the ω3/ω6 ratio in seafood and food in general [32] and its strong relationship with human health. To prevent neurological, inflammatory, and cardiovascular disorders, the World Health Organization (WHO) currently recommends that this ratio should be higher than 0.1 in the human diet [33]. Additionally, the European Nutritional Society reported that a human diet with an ω3/ω6 ratio of 1/5 or higher would have health benefits [34]. Average values obtained in the present study (Figure 2) are included in the 7.0–8.6 range. Notably, the highest ω3/ω6 ratio was found for sardines corresponding to a 0-day storage, while the lowest was obtained in sardines previously stored for 15 days; differences between both kinds of samples were found significant (*p* < 0.05). Therefore, an increased previous holding time has led to a higher loss of ω3 FA than for ω6 FA. However, and in spite of this decrease, ω3/ω6 ratio values in all canned samples can be considered highly nutritional according to the abovementioned health requirements for this FA ratio.

### 3.3. Assessment of Triacylglycerol (TAG) and Diacylglycerol (DAG) Values

Values detected for TAG and DAG lipid classes are reported in Table 1. TAG showed to be the most abundant lipid class in the lipid fraction, values being included in the 618.8–678.1 g·kg^−1^ lipid range. Average values of TAG content showed a similar trend with previous holding time than lipid content. Thus, an increasing tendency was detected for samples corresponding to the 0–10-day period, while a decreasing trend was obtained in canned sardines of the 13–15-day storage time. As a result, a higher (*p* < 0.05) TAG content was detected in sardines chilled for 10 days than in fish corresponding to 13 and 15 days of storage.

DAG contents were included in the 11.9–43.4 g·kg^−1^ lipid range. An evolution opposite that for TAG class was detected with storage time. Thus, an accurate inverse correlation value was observed between both lipid class values (*r* = −0.81). For average DAG content, slight differences were detected among samples corresponding to the 0–10-day period; in contrast, average values showed a marked increase when considering canned muscle corresponding to 13 and 15 days of storage. Remarkably, the highest DAG value (*p* < 0.05) was detected in fish corresponding to day 15 in agreement with the lowest TAG content found in such kind of canned samples.

Present data obtained for TAG and DAG in the different canned samples can be considered the result of different factors. On one side, TAG hydrolysis is reported to be produced during storage on ice as a result of endogenous and microbial enzyme activity [10,12] as well as during canning as a result of the thermal treatment [7]; this would lead to a TAG content decrease and to a DAG content increase. Additionally, thermal treatment itself would produce lipid breakdown so that general losses of the content of most lipid classes may occur [4,6]. Notably, small-size molecules are known to react easier according to the fact that they imply a lower steric hindrance [35]; this would mean that this broken effect during heating would be more important in DAG than in TAG and lead to important losses in DAG value. Furthermore, small-size molecules and polar lipids produced as a result of lipid hydrolysis or breakdown are likely to be extracted by the hydrophilic filling medium employed and be lost from the fish muscle. According to such different effects, a general trend of TAG and DAG values could not be detected in current samples corresponding to the 0–15-day period. In contrast, definite tendencies were detected by considering two different storage periods separately (i.e., 0–9-day and 13–15-day periods).

Concerning the importance of lipid hydrolysis development, it is generally accepted that accumulation of partially hydrolysed acylglycerols resulting from lipid hydrolysis in fish muscle has no nutritional significance [8]. Nevertheless, this damage pathway has been recognised as a most important event during fish processing as leading to deteriorative changes of muscle texture, acceleration of lipid oxidation compound formation, and off-odour and off-taste development [36,37].

Related to lipid hydrolysis development, previous research has addressed the formation of free fatty acid (FFA) compounds in canned fish that was previously subjected to storage on ice. Thus, a progressive increase of FFA content was observed in brine-canned sardines (*S. pilchardus*) by increasing the previous storage time on ice [15]. Later on, Rodríguez et al. [17] observed an increased FFA content in sunflower oil-canned salmon (*Oncorhynchus kisutch*) that had been stored under common ice and slurry ice conditions; remarkably, this increase was found higher by increasing the storage time. Recently, increased FFA values were obtained in brine-canned Chub mackerel (*Scomber colias*) by increasing the previous storage time up to 9 days [14].

### 3.4. Determination of Browning Development

**Figure 3:**
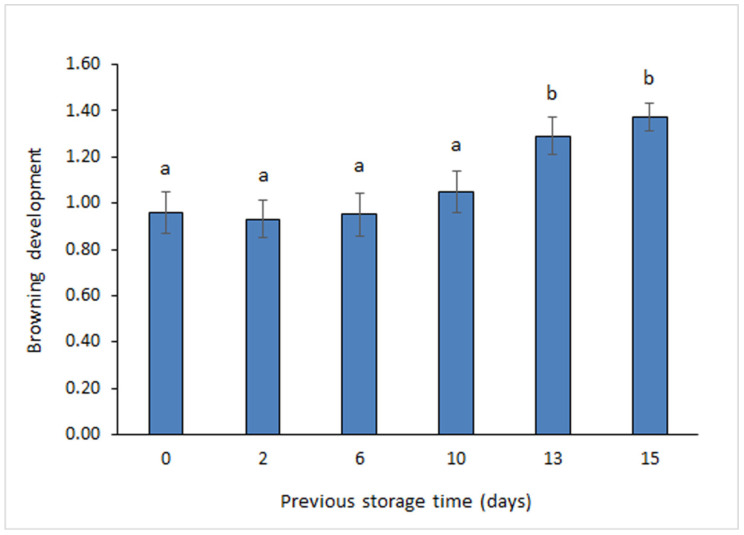
Determination of the browning development in canned sardines muscle subjected previously to storage on ice. Average values of three replicates (*n* = 3). Standard deviations are indicated by bars. Average values accompanied by different letters (a,b) denote significant differences (*p* < 0.05).

Results on browning development in canned muscle are depicted in Figure 3. Browning detection did not provide differences (*p* > 0.05) in canned sardines corresponding to the 0–10-day period. However, a marked increase (*p* < 0.05) was detected when taking into account canned samples corresponding to 13 and 15 days of storage. Therefore, an increasing effect on the development of browning reaction could be concluded by increasing the previous storage time (*r* = 0.92). Additionally, an accurate correlation value of browning development was proved with DAG content (*r* = 0.84) and with PUFA/STFA ratio (*r* = −0.84).

As previously mentioned, a marked lipid oxidation development has been reported in fish muscle during storage on ice [12,31]. This damage mechanism has shown an increasing significance with holding time [8]. The resulting decrease of lipid stability in raw material would enhance the lipid oxidation development during the subsequent thermal treatment (i.e., cooking and sterilisation) of the canning process [14,16]. Thus, the strong heat treatment and the presence of some catalysts (namely, metalloproteins) in the fish muscle can favour non-enzymatic lipid oxidation and hydrolysis so that detrimental flavour and essential nutrient losses can be produced [12,38]. Precursors of brown substances are lipophilic substances that are both formed and decomposed especially during the thermal treatment of the canning process. Notably, prominent changes occurred by discolouration as oxidation products resulting from highly polyunsaturated fish lipids give intensive colour reaction with basic groups of fish muscle proteins (i.e., amino acids, peptides, phospholipids, etc.) [39,40,41].

Previous research accounts for the assessment of interaction compounds (i.e., oxidised lipids and protein-type molecules) by detection of the fluorescent properties of canned fish muscle. Thus, Aubourg and Medina [15] found an increased formation of fluorescent compounds in brine-canned sardines (*S. pilchardus*) that were previously subjected to storage on ice; remarkably, a direct effect of holding time was proved on fluorescent compound formation. Later on, an increased formation of fluorescent compounds in oil-canned sardines (*S. pilchardus*) was proved by increasing the storage time on ice (from 0 to 5 days) [16]; notably, this increase was detected both in the canned muscle as in the packing sunflower oil. Additionally, Rodríguez et al. [17] studied the effect of ice as previous slaughter and storage conditions (i.e., storage up to 9 days) for salmon (*O. kisutch*); as a result, an increased holding time led to a higher b* colour parameter in oil-canned muscle, closely related to the browning development assessment. Recently, the effect of a prior storage period on ice (0–9-day storage) on the quality of brine-canned Chub mackerel (*S. colias*) was investigated [14]; as a result, an increased storage time led to higher values of thiobarbituric acid index and fluorescent compound formation.

### 3.5. Sensory Evaluation

Sensory acceptance of canned sardines was analysed by odour, appearance, and texture descriptors (Table 3). For all descriptors, average values corresponding to the highest quality degree were detected in samples corresponding to 6 and 10 days of previous storage; however, significant differences (*p* < 0.05) were very scarce among samples corresponding to the 0–10-day previous storage. Furthermore, average values corresponding to the lowest quality were detected in canned samples subjected previously to 13-day and 15-day storage. Consequently, a negative effect on sensory acceptance could be concluded if advanced storage times were employed. Remarkably, scores obtained for appearance (samples corresponding to 15-day storage) and texture (samples corresponding to 13-day and 15-day storage) were not considered acceptable by the sensory panel. Otherwise, samples previously subjected to 0–10-day holding times were considered acceptable.

Accurate correlation values of sensory descriptors with chemical parameters were observed. Thus, off-odour detection revealed a direct relationship with DAG content (*r* = 0.85), but inverse with the TAG content (*r* = 0.89). Concerning quality loss by appearance assessment, this descriptor also led to direct and inverse relationships with DAG (*r* = 0.92) and TAG (*r* = −0.85) values, respectively. These relationships agree with previous research showing the relevant effect of lipid hydrolysis development on off-odour development and breakdown of fish muscle [17,36,38]. Texture quality loss showed valuable correlation values with browning (*r* = 0.90) and DAG (*r* = 0.89) values and fair relationships with the PUFA/STFA ratio (*r* = −0.78) and the TAG (*r* = −0.78) content. A strong relationship of texture quality loss with lipid hydrolysis and oxidation has already been observed in canned fish [17,36,38]. Additionally, texture has been reported to be damaged by reaction of proteins and oxidised lipids [8,39].

Previous research has addressed the effect of previous holding conditions on the sensory quality of the corresponding canned fish food. Thus, a lower acceptance (appearance, colour, odour, taste, and texture) was produced by delayed previous icing (i.e., storage at 28 ± 2 °C for about six hours) of white sardines (*Kowala coval*) [13]. Later on, Jeya Shakila et al. [42] found that holding tuna (*Katsuwonus pelamis*), seerfish (*Scomberomorus commersonii*), and sardines (*Sardinella gibbosa*) destined to canning at 30 ± 2 °C for six hours led to canned products that were safe and sensory acceptable. Losada et al. [16] showed a firmness increase and a cohesivity decrease in oil-canned sardines (*S. pilchardus*) by increasing the previous storage time on ice from 0 to 5 days. Furthermore, Rodríguez et al. [17] studied the effect of flake ice as previous storage up to 9 days for salmon (*O. kisutch*); an increased holding time led to higher firmness and lower cohesivity values of oil-canned muscle as well as to increased oxidised odour.

## 4. Conclusions

A substantial effect of previous holding time on lipid quality and sensory acceptance was detected in canned sardines. Thus, an increased storage time on ice led to a significant (*p* < 0.05) decrease of PUFA/STFA (10–15-day period) and ω3/ω6 (15 days) ratios as well as to a relevant (*p* < 0.05) development of browning (13–15-day period). Furthermore, sensory evaluation of appearance and texture indicated that the longest holding times tested (15 days for appearance; 13 and 15 days for texture) led to canned products with decreased quality; remarkably, canned samples subjected previously to such storage conditions were not considered acceptable by the sensory panel.

On the basis of the present results, it is recommended to minimise the time elapsed between on-board handling or post-harvest handling and cooling steps. Additionally, raw material should be delivered as soon as possible into the factory without breaking the cold chain. Once in the cannery, efficient storage techniques for the refrigeration of marine species material ought to be employed. If relatively long previous storage times (i.e., over 6–10 days) or high storage temperatures (i.e., over 0–2 °C) are required, fish ought to be subjected to advanced technologies susceptible to partially inhibit the microbial and endogenous enzyme activity and lipid oxidation development during the holding period. For it, previous physical (i.e., high-hydrostatic pressure, preserving packing, irradiation in general) or chemical (i.e., preservative compound addition) treatment could be an appropriate tool to be applied.

## Figures and Tables

**Table 1:** Chemical analysis of canned sardines subjected to storage on ice before canning.

Chemical Determination	Previous Storage Time on Ice (Days)					
	0	2	6	10	13	15
Moisture (g·kg^−1^ muscle)	714.6 ± 8.5 ^c,d^	711.1 ± 8.4 ^b,c,d^	698.8 ± 5.6 ^a,b^	687.8 ± 8.1 ^a^	701.6 ± 12.0 ^a,b,c^	725.2 ± 3.7 ^d^
Lipids (g·kg^−1^ muscle)	48.8 ± 2.8 ^a^	53.9 ± 9.6 ^a,b^	60.1 ± 4.2 ^a,b^	68.4 ± 7.2 ^b^	62.4 ± 10.2 ^a,b^	61.7 ± 14.3 ^a,b^
Triacylglycerols (g·kg^−1^ lipids)	642.4 ± 12.5 ^a,b^	654.7 ± 24.6 ^a,b,c^	661.7 ± 15.4 ^b,c^	678.1 ± 4.7 ^c^	647.0 ± 4.7 ^a,b^	618.8 ± 35.7 ^a^
Diacylglycerols (g·kg^−1^ lipids)	13.3 ± 1.0 ^a^	12.4 ± 6.7 ^a^	12.4 ± 5.3 ^a^	11.9 ± 7.9 ^a^	21.9 ± 7.5 ^a^	43.4 ± 7.0 ^b^

Average values of three replicates (*n* = 3) ± standard deviations; in each row, values followed by different letters (a–d) denote significant differences (*p* < 0.05).

**Table 2:** Fatty acid (FA) analysis (g·100 g^−1^ total FA) of canned sardines subjected to storage on ice before canning.

FA or FA Group	Previous Storage Time on Ice (Days)					
	0	2	6	10	13	15
14:0	1.0 ± 0.3	1.2 ± 0.2	0.9 ± 0.4	1.3 ± 0.5	1.3 ± 0.3	1.8 ± 0.5
15:0	0.4 ± 0.1	0.3 ± 0.1	0.4 ± 0.1	0.4 ± 0.1	0.3 ± 0.1	0.5 ± 0.2
16:0	27.1 ± 1.5	27.5 ± 1.1	27.2 ± 1.2	27.6 ± 1.9	28.3 ± 2.1	28.4 ± 1.4
17:0	0.4 ± 0.1	0.5 ± 0.1	0.4 ± 0.2	0.4 ± 0.1	0.5 ± 0.2	0.5 ± 0.1
18:0	3.6 ± 0.8	4.0 ± 0.5	3.6 ± 1.0	4.1 ± 1.1	4.1 ± 0.8	4.6 ± 0.9
24:0	0.2 ± 0.1	0.3 ± 0.1	0.1 ± 0.0	0.2 ± 0.0	0.2 ± 0.1	0.3 ± 0.0
16:1 ω7	2.3 ± 0.4	2.4 ± 0.7	2.5 ± 0.7	2.2 ± 0.6	2.2 ± 0.6	2.4 ± 0.5
18:1 ω9	5.6 ± 0.7	5.7 ± 0.9	5.9 ± 0.8	5.5 ± 0.8	5.6 ± 0.3	5.8 ± 0.7
18:1 ω7	2.0 ± 0.3	1.8 ± 0.3	2.1 ± 0.6	1.8 ± 0.7	2.0 ± 0.2	2.0 ± 0.8
20:1 ω9	1.7 ± 0.7	1.5 ± 0.9	1.8 ± 0.4	1.7 ± 0.4	1.7 ± 0.7	1.8 ± 0.8
22:1 ω11	0.3 ± 0.1	0.2 ± 0.2	0.3 ± 0.2	0.3 ± 0.1	0.3 ± 0.1	0.4 ± 0.2
18:2 ω6	1.0 ± 0.4	1.6 ± 0.3	1.2 ± 0.7	1.4 ± 0.6	1.3 ± 0.6	1.5 ± 0.5
20:4 ω6	4.7 ± 0.7	4.5 ± 0.9	4.6 ± 0.8	4.8 ± 0.8	4.3 ± 0.6	5.2 ± 0.7
18:3 ω3	0.6 ± 0.2	0.6 ± 0.2	0.5 ± 0.1	0.7 ± 0.4	0.7 ± 0.1	0.5 ± 0.2
18:4 ω3	0.9 ± 0.1	1.0 ± 0.3	1.0 ± 0.2	0.9 ± 0.3	0.8 ± 0.3	0.8 ± 0.2
20:5 ω3 (EPA)	12.8 ± 1.3	11.9 ± 1.1	12.4 ± 1.1	11.7 ± 1.7	11.5 ± 2.1	11.3 ± 1.9
22:5 ω3	0.8 ± 0.1	0.8 ± 0.3	0.9 ± 0.3	1.0 ± 0.2	1.0 ± 0.2	0.8 ± 0.4
22:6 ω3 (DHA)	33.7 ± 3.1	33.0 ± 4.1	33.3 ± 3.7	33.1 ± 3.7	32.8 ± 5.1	33.2 ± 3.0
Total STFA	32.7 ± 3.2 ^b^	33.8 ± 3.0 ^b^	32.6 ± 2.7 ^b^	34.0 ± 3.9 ^b^	34.7 ± 3.7 ^b^	35.1 ± 3.0 ^b^
Total MUFA	11.9 ± 2.2 ^a^	11.6 ± 2.0 ^a^	12.6 ± 2.3 ^a^	11.5 ± 1.6 ^a^	11.8 ± 1.7 ^a^	10.4 ± 2.2 ^a^
Total PUFA	54.5 ± 5.2 ^c^	53.4 ± 4.2 ^c^	53.9 ± 5.3 ^c^	53.6 ± 5.5 ^c^	52.4 ± 4.9 ^c^	52.3 ± 4.7 ^c^

Average values of three replicates (*n* = 3) ± standard deviations; values for individual FA and FA groups did not show significant differences (*p* > 0.05) as a result of the previous storage time. In each column, average values of FA groups followed by different letters (a–c) denote significant differences (*p* < 0.05). Abbreviations employed: STFA (saturated FA); MUFA (monounsaturated FA); PUFA (polyunsaturated FA); EPA (eicosapentaenoic acid), and DHA (docosahexaenoic acid).

**Table 3:** Sensory acceptance of canned sardines subjected previously to storage on ice **.

Descriptor	Previous Storage Time on Ice (Days)					
	0	2	6	10	13	15
Appearance	43.0 ± 9.0 ^a^	39.7 ± 6.0 ^a^	34.0 ± 6.1 ^a^	35.0 ± 2.0 ^a^	44.0 ± 11.4 ^a^	72.7 ± 14.6 ^b^
Odour	35.0 ± 2.7 ^b^	31.0 ± 5.3 ^a,b^	26.3 ± 5.9 ^a,b^	26.3 ± 3.8 ^a^	35.7 ± 7.4 ^a,b^	44.0 ± 6.2 ^b^
Texture	43.1 ± 1.3 ^a^	40.9 ± 2.7 ^a^	38.4 ± 4.8 ^a^	40.5 ± 1.6 ^a^	52.4 ± 3.8 ^b^	59.1 ± 6.1 ^b^

Average values of three replicates (*n* = 3) ± standard deviations. For each descriptor, values followed by different letters (a,b) denote significant differences (*p* < 0.05).

## Data Availability

Not applicable.
